# Reduced arctic tundra productivity linked with landform and climate change interactions

**DOI:** 10.1038/s41598-018-20692-8

**Published:** 2018-02-05

**Authors:** Mark J. Lara, Ingmar Nitze, Guido Grosse, Philip Martin, A. David McGuire

**Affiliations:** 1 Department of Plant Biology, University of Illinois Urbana-Champaign, Urbana, Illinois 61801 USA; 2 Institute of Arctic Biology, University of Alaska Fairbanks, Fairbanks, Alaska 99775 USA; 3 Alfred Wegener Institute Helmholtz Centre for Polar and Marine Research, Periglacial Research Unit, 14473 Potsdam, Germany; 4 Institute of Earth and Environmental Science, University of Potsdam, 14476 Potsdam, Germany; 5 U.S. Fish and Wildlife Service, Fairbanks, Alaska 99701 USA; 6 U.S. Geological Survey, Alaska Cooperative Fish and Wildlife Research Unit, University of Alaska Fairbanks, Fairbanks, Alaska 99775 USA

## Abstract

Arctic tundra ecosystems have experienced unprecedented change associated with climate warming over recent decades. Across the Pan-Arctic, vegetation productivity and surface greenness have trended positively over the period of satellite observation. However, since 2011 these trends have slowed considerably, showing signs of browning in many regions. It is unclear what factors are driving this change and which regions/landforms will be most sensitive to future browning. Here we provide evidence linking decadal patterns in arctic greening and browning with regional climate change and local permafrost-driven landscape heterogeneity. We analyzed the spatial variability of decadal-scale trends in surface greenness across the Arctic Coastal Plain of northern Alaska (~60,000 km²) using the Landsat archive (1999–2014), in combination with novel 30 m classifications of polygonal tundra and regional watersheds, finding landscape heterogeneity and regional climate change to be the most important factors controlling historical greenness trends. Browning was linked to increased temperature and precipitation, with the exception of young landforms (developed following lake drainage), which will likely continue to green. Spatiotemporal model forecasting suggests carbon uptake potential to be reduced in response to warmer and/or wetter climatic conditions, potentially increasing the net loss of carbon to the atmosphere, at a greater degree than previously expected.

## Introduction

Over the past few decades, greening or increased vegetation productivity in Arctic tundra lowlands has been inferred from trends in satellite-derived Normalized Difference Vegetation Index (NDVI)^1–3^. Researchers have speculated that these positive NDVI trends may be in response to reduced snow cover or warming, which may manifest on the landscape in the form of shrubification^4^, increased vegetation biomass (height, length, density)^3,5^, changing phenoperiods^6^, or increased surface water associated with thermokarst^7^. However, others have hypothesized that greening trends may have been in response to summer sea ice retreat^8^ or increased rates of infrastructure development associated with oil drilling and exploration^9^. It is important to recognize that arctic tundra landscapes are highly heterogeneous and have historically been in a slow but continuous state of change associated with permafrost thaw and aggradation processes related to periglacial landscape dynamics^10^. Consequently, patches of browning or negative NDVI trends have also been commonly observed across arctic tundra regions, but until recently the greening signal prevailed^11^. Since 2011, regional shifts toward surface browning have reversed the direction of the trend after nearly 33 years of arctic greening^11^. If such change in greening indeed corresponds with a reduction in vegetation productivity or carbon uptake capacity via photosynthesis, then nearly all ecosystem and earth system models have not foreseen this shift. Therefore, it is urgent to understand what may be controlling this spatiotemporal shift in browning across the Arctic and if this change is anomalous or represents a new long-term trajectory towards reduced vegetation productivity and carbon uptake in the Arctic tundra^12^.

To date, our knowledge of circumpolar patterns of greening are derived from coarse-resolution sensors on board satellites, such as the global 8 km and 1 km resolution Advanced Very High Resolution Radiometer (AVHRR) and to a lesser extent the 1 to 0.25 km resolution Moderate Resolution Imaging Spectroradiometer (MODIS)^1,3^, all of which robustly produce spectral observations spanning gradients of time and space. However, data products generated by these observation platforms are limited in their ability to evaluate meso- to fine-scale patterns that may control greening and browning at coarser resolutions^11^. Although, notable progress in the evaluation of landscape-level patterns of Arctic NDVI at fine-scales has been made^9,13–17^, these studies typically feature a similar physiography and/or climate, limiting the evaluation of how similar vegetation types and/or landforms may respond to different climate regimes. It is difficult to assess such patterns across Arctic regions for a variety of reasons, but perhaps most importantly because of the limited availability of high quality land cover datasets, which do not exist, are only regionally specific, or do not adequately represent heterogeneity across the tundra landscape to decipher meaningful patterns^18^. Thus, without adequate spatial coverage and resolution of land cover data products used to link plot to landscape-level datasets, our ability to monitor and interpret patterns of change in the Arctic remains severely limited^18^.

**Figure 1:**
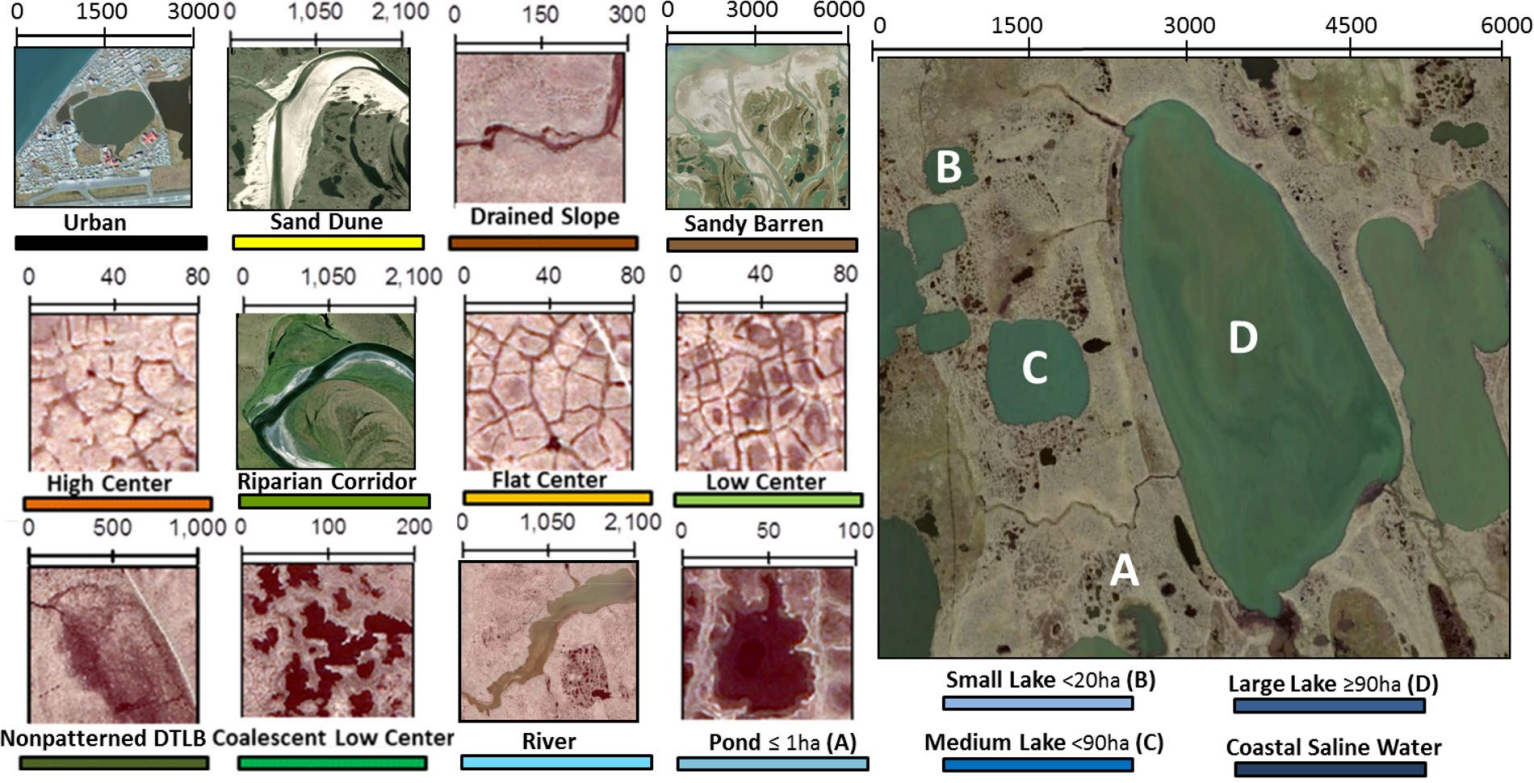
Dominant geomorphic types on the Arctic Coastal Plain of northern Alaska, as observed from high resolution satellite imagery^44^ (copyright DigitalGlobe, Inc.). Figure created in Esri ArcMap 10.4.

The Arctic Coastal Plain (ACP) of northern Alaska represents an expansive geographic region of tundra where decadal trends in greening have recently strengthened^1,2,11^, yet the magnitude of change varied nearly two-fold between the western and eastern ACP (i.e. eastern Chukchi and Beaufort, respectively)^11^. Concurrently, the climate of the ACP has changed and appears to be regionally specific, with some regions warming more extensively than others and increasing/decreasing in precipitation. Additionally, across the ACP, the spatial distribution of fine-scale tundra landforms (here after referred to as “geomorphic types”), varies significantly^19–21^, likely with different sensitivities to climate variability and change^12,22^. Here we focus on evaluating key factors controlling decadal scale surface NDVI (i.e. greening versus browning), and evaluate what regions and geomorphic types are most sensitive to future climate change. We calculated decadal NDVI trends using Landsat imagery from sensors, Thematic Mapper (TM), Enhanced Thematic Mapper Plus (ETM+), and the Observing Land Imager (OLI), and assessed the variability in greenness from local to regional scales by using novel mapping techniques^19^ to create a polygonal tundra map (30 × 30 m resolution), which represents fifteen of the most dominant tundra geomorphic types (e.g., high/low-center polygon; Fig. 1), nested within regional watersheds^23^, and ecological landscapes^20^ spanning the ACP.

## Methods

We studied the effects of climate and tundra geomorphic types on decadal scale trends in greening across the ACP, which stretches from the western coast along the Chukchi sea to the Beaufort coastal plains at the Alaskan/Canadian border (latitude: 68–71°N; longitude: 140–167°W). This region is representative of ~1.9 million km^2^ of Arctic coastal tundra^24^, characterized by low topographic relief, with abundant ice wedge polygons^25^, thick permafrost^20^, and predominantly wet sedge or herbaceous vegetation^21^. Summer and winter temperatures range from 5 to 15 °C and −18 to −40 °C, respectively (www.ncdc.noaa.gov). Annual precipitation is variable but typically ranges from 120–200 mm. We defined the ACP spatial domain by the geographic land area within the Northern/Southern Chukchi Sea Coast, Beaufort Sea Coast, Beaufort Coastal Plain, and a section of the Brooks Range Foothills on the Krusenstern Coastal Plain, all of which are composed of a high density of polygonal tundra or patterned ground (Fig. 1).

We expand upon a novel automated object based image analysis (OBIA) geomorphic mapping approach^19^, for characterizing tundra geomorphology across the ACP (58,691 km^2^). The initial application of tundra mapping was developed for a polygonal coastal tundra ecosystem on the Barrow Peninsula (1800 km^2^)^19^. Twelve LandSat-8 OLI (summer) satellite images (Supplemental Table S1) were processed and mosaicked within ArcGIS^TM^ 10.4 (ESRI) for tundra geomorphology mapping. An OBIA land cover classifier (eCognition^TM^ v.9.1, Trimble) was parameterized using various rules, thresholds, spectral indices, and proximity functions to differentiate between geomorphic types^26^. Multiresolution segmentation and spectral difference algorithms were used to separate pixels into “image objects”, which were divided into open water, aquatic, wet, moist, dry classes using reference data (i.e. field/ground truth points and high resolution aerial/satellite imagery) and class thresholds based on Normalized Difference Water Index (NDWI).

**Figure 2:**
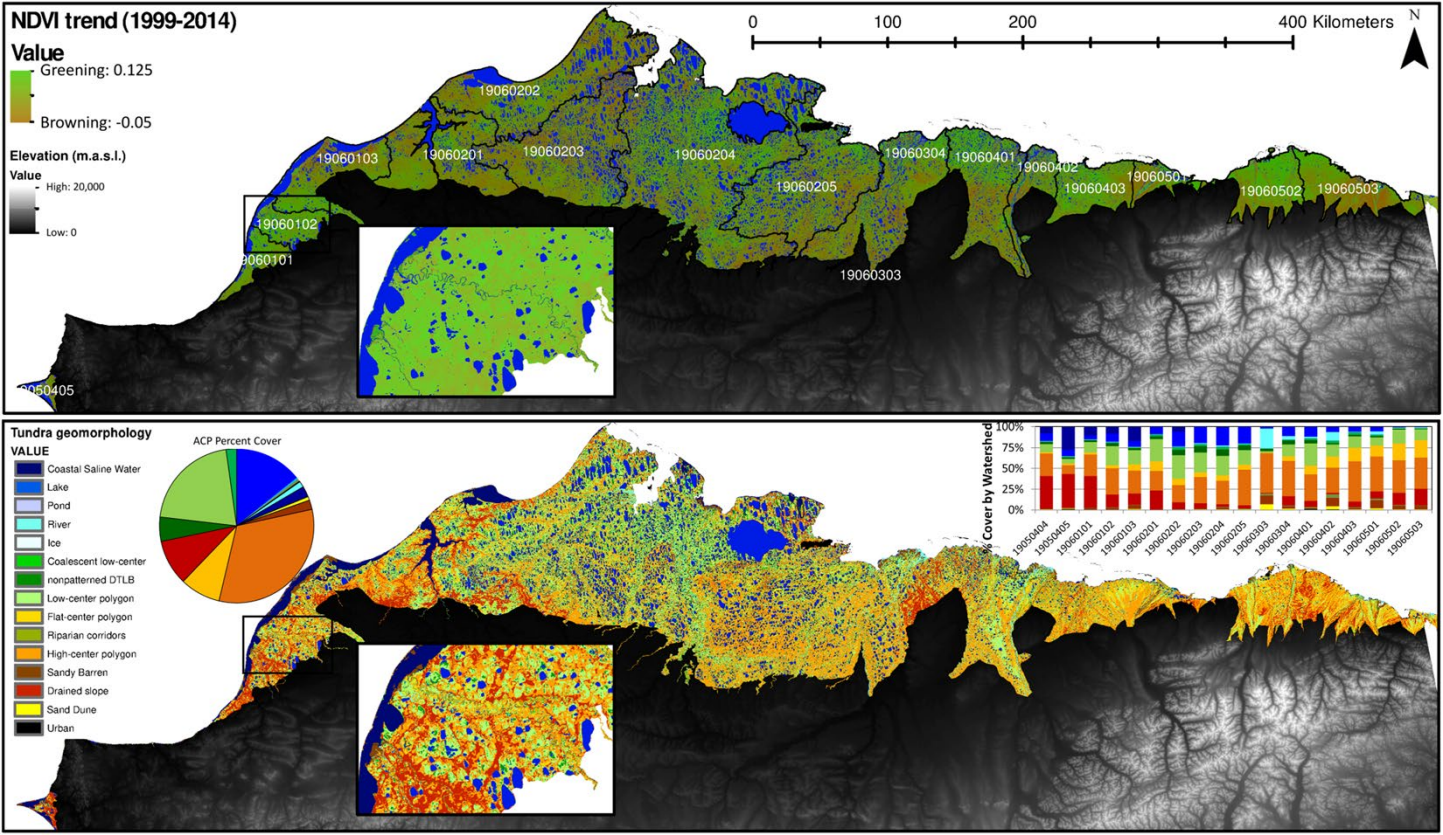
Decadal time scale Landsat derived greenness (NDVI) trend and regional watersheds (top panel), and tundra geomorphology map (bottom panel). Tundra geomorphology map was developed at a 30 × 30 m spatial resolution (see methods for more details). Note the variability in geomorphology distribution associated with regional watersheds (stacked bar chart). Greenness Trend map was created following established workflows^1^, while tundra geomorphology map was created in Trimble eCognition v.9.1, both maps projected in Esri ArcMap 10.4.

A series of proceeding functions were developed using individual and combined spectral bands, geometric object shapes/sizes (i.e. perimeter, area, roundness), and proximity functions to further differentiate respective geomorphology classes^26^. Using this approach we mapped fifteen geomorphological and hydrologically distinct geomorphic types (Figs 1 and 2) at 30 × 30 m spatial resolution, including (qualitatively ranked from wet to dry), 1) coastal saline water, which commonly encroach into terrestrial lakes due to processes related to coastal erosion or lagoon formation, 2) lakes (large: >90 ha, medium: ≤90 and >20 ha, small: ≤20 ha), 3) rivers, 4) ponds, 5) coalescent low-center polygons, 6) nonpatterned drained thaw lake basins, 7) low-center polygons, 8) flat-center polygons,9) riparian corridors 10) high-center polygons, 11) sandy barrens, 12) drained slopes, 13) sand dunes, 14) ice/snow, and 15) urban. Refer to Supplemental Table S2 for surface characteristics related to moisture, relief, and vegetation communities specific to geomorphic type. For this analysis, all lake sizes were grouped into one “Lake” category.

The tundra geomorphic map (Fig. 2) was validated using an array of oblique aerial/ground based photography^21^ and 249 high resolution (2.5 m resolution) SPOT-5 orthorectified image tiles covering >80% of the ACP (Supplemental Table S3; gina.alaska.edu)^26^. We used a stratified random sampling design, where 1000 reference sites were used to evaluate map accuracy within both the Arctic Peaty Lowlands and the Arctic Sandy Lowlands^20^.

We computed and analyzed NDVI trends using Landsat imagery following workflows developed for the Siberian, Lena River Delta^13^, where each pixel has a temporal coverage of 40 to 110 observations collected between 1999–2014^26^. Throughout this paper, we refer to greening and browning as increased and decreased NDVI, respectively. Prior to data extraction from NDVI maps (Fig. 2), all coastal saline water, lakes, rivers, and urban pixels were removed. Primarily due to limited data acquisition across the ACP prior to 1999, we restrict decadal greenness trends (i.e. absolute change and percent change relative to 1999) to 1999–2014 throughout this paper.

Due to our use of multiple Landsat sensors (i.e. TM, ETM+, and OLI) within the NDVI trend map product, we calculated the sensor bias of NDVI at three different locations across Alaska, each containing a sample of 40,000 pixels^26^. We found minor calibration differences between sensors (i.e. one percent of the signal), while sensor specific NDVI distributions were consistent^26^.

Two data subsets were extracted from decadal NDVI products (i.e. absolute and percent change), where local to regional absolute and percent change in greenness was extracted for each geomorphic type in each Hydrological unit code 8 (HUC 8) watershed (Supplemental Fig. S4, Supplemental Table S5), for use in boosted regression tree (BRT)^27^ and multivariate regression model analysis (Supplemental Table S6), respectively. Hydrological unit code 8 watersheds were computed by the Alaska Watershed and Stream Hydrography Enhanced Dataset Project^23^, where each watershed was divided into two subunits (i.e. lowland/upland) associated with the second elevation quantile. Prior to spatial analysis, we identified outlier pixels by determining if NDVI change was >75.0% or < −75.0% per decade, and used a nearest neighbor filtering algorithm for the recalculation of pixels within a 5 × 5 pixel window, which represented <0.1% of all pixels on the ACP (residual unfiltered open water pixels).

Datasets used in all analyses included the following predictor variables: elevation (60 m resolution), climate *normals* (1960–1999), *change* (difference between 2000–2010 and “normals”), and *anomalies* (“change”/“normal”) for annual temperature, precipitation, and potential evapotranspiration downscaled to 771 m resolution by the Scenarios Network for Alaska and Arctic Planning^28^. In addition, we calculated the percent cover of soil moisture regime for each 771 × 771 m pixel (resolution standardized with input climate data), estimated by associations between geomorphic type and field observations^19,22,29^. We combined geomorphic classes into their respective moisture categories as follows: *open water* (coastal saline water, lakes, rivers), *aquatic* (ponds, coalescent low-center polygons), *wet* (nonpatterned drained thaw lake basins, low-center polygons), *moist* (flat-center polygons, riparian corridors), *dry* (high-center polygons, drained slopes) and *other* (sandy barrens, sand dunes, ice/snow, urban).

The TreeNet Gradient Boosting machine, developed within the Salford Predictive Modeler^TM^ v.8.0 (Salford Systems), was used to run all BRT analyses. A “shaving” procedure was used to iteratively remove and rerun the BRT analysis to minimize the mean squared error, where 80% (n = 276) of the dataset was used for model development (i.e. learning) and 20% (n = 53) was used for independent model evaluation (i.e. testing). BRT learning rate, tree complexity, and loss criterion, was set to 0.1, 6, and Huber-M, respectively. To ensure reproducibility, we used a seed state of 987654321 for model initialization. Partial dependency plots were used to show the response of individual predictor variables to the BRT analysis, using fitted functions^27^. Fitted functions detail the effect of a variable on the response after accounting for average effects of all other variables in the model^27^.

Stepwise multivariate regression and Pearson’s correlation analyses were ran in Jmp Pro^TM^ v.10 (SAS). Input datasets used in the stepwise procedure (Supplemental Table S7) were all transformed to fit the assumptions of normality, and only important factors identified by the preceding BRT analysis were input into the stepwise procedure, used to predict regional greenness trends by fitting potentially important climate and/or environmental variables. A five-fold cross validation was concurrently performed, which divided the dataset into 5 subsets or 80:20 and iteratively used each 80% subset to predict the other 20% (e.g. k-1). The average R^2^ of all models was then calculated. Multivariate regression models were used to forecast change in the NDVI over the next decade (i.e. 2020–29), forced by the top five climate models to accurately represent Arctic and Alaskan regions^28,30^ used in the IPCC Fifth Assessment Report (Table 1). These included the Community Earth System Model 4 (NCAR-CCSM4), Coupled Model 3.0 (GFDL-CM3), ModelE/Russell (GISS-E2-R), Institut Pierre-Simon Laplace Coupled Model v5A (IPSL-CM5A), and the Coupled General Circulation Model v3.0 (MRI-CGCM3). Selected model runs included the AR5 representative concentration pathways RCPs 8.5 (high) and 4.5 (low). We assumed no change in tundra geomorphic type distribution for model simulations.

**Table 1:** Projected change in precipitation and temperature (2020–2029) relative to climate normals for the Arctic Coastal Plain of northern Alaska by climate model and RCP emission scenario. Models are generally listed from lowest to highest projected change.

	Climate Model	RCP 4.5		RCP 8.5	
		Mean	Stdev	Mean	Stdev
Temperature change (°C)	MRI-CGCM3	1.37	0.22	1.21	0.18
	GISS-E2-R	1.4	0.767	1.94	0.114
	IPSL-CM5A	1.62	0.129	3.45	0.2
	NCAR-CCSM4	2.93	0.19	2.56	0.2
	GFDL-CM3	5.46	0.58	4.63	0.44
Precipitation change (mm)	MRI-CGCM3	9.27	15.96	17.18	10.28
	GISS-E2-R	31.27	10.31	16.52	11.98
	IPSL-CM5A	35.15	11.15	9.48	7.93
	NCAR-CCSM4	14.85	8.22	28.4	10.19
	GFDL-CM3	46.6	19.99	45.47	9.45

Temp. Normal (1960–1999): −11.51 °C, ±0.93.

Precip. Normal (1960–1999): 236.63 mm, ±21.29.

## Results

We found the regional distribution of tundra geomorphic types and greenness to vary markedly across the Arctic Coastal Plain of northern Alaska (Fig. 2, Supplemental Fig. S4). The newly developed tundra geomorphology map represented the spatial distribution of polygonal tundra well with an overall map accuracy and Cohen’s Kappa coefficient of 76% and 0.73, respectively (Supplemental Table S3)^26^. Map statistics indicated that high-center polygons, low-center polygons, and lakes were the predominant features across the ~60,000 km² ACP representing 69.3% of the total land cover area (Fig. 2). Watersheds ranged from 15 to 13,406 km² with a median of 2,128 km², which also varied in geomorphic type distribution (Fig. 2, Supplemental Fig. S4). Historical NDVI trends varied between regional watersheds (Supplemental Fig. S4), and across geomorphic types (Supplemental Tables S5 and S6), suggesting that indeed trends in greening and browning are locally and regionally specific. Generally, across the ACP historical trends in NDVI (±standard deviation) differed by geomorphic type as observed in ponds (0.005 ± 0.22), coalescent low-center (0.035 ± 0.11), nonpatterned drained thaw lake basins (0.032 ± 0.14), low-center (0.044 ± 0.06), flat-center (0.046 ± 0.05), riparian corridor (0.039 ± 0.13), high-center (0.041 ± 0.05), and drained slope (0.042 ± 0.04).

**Figure 3:**
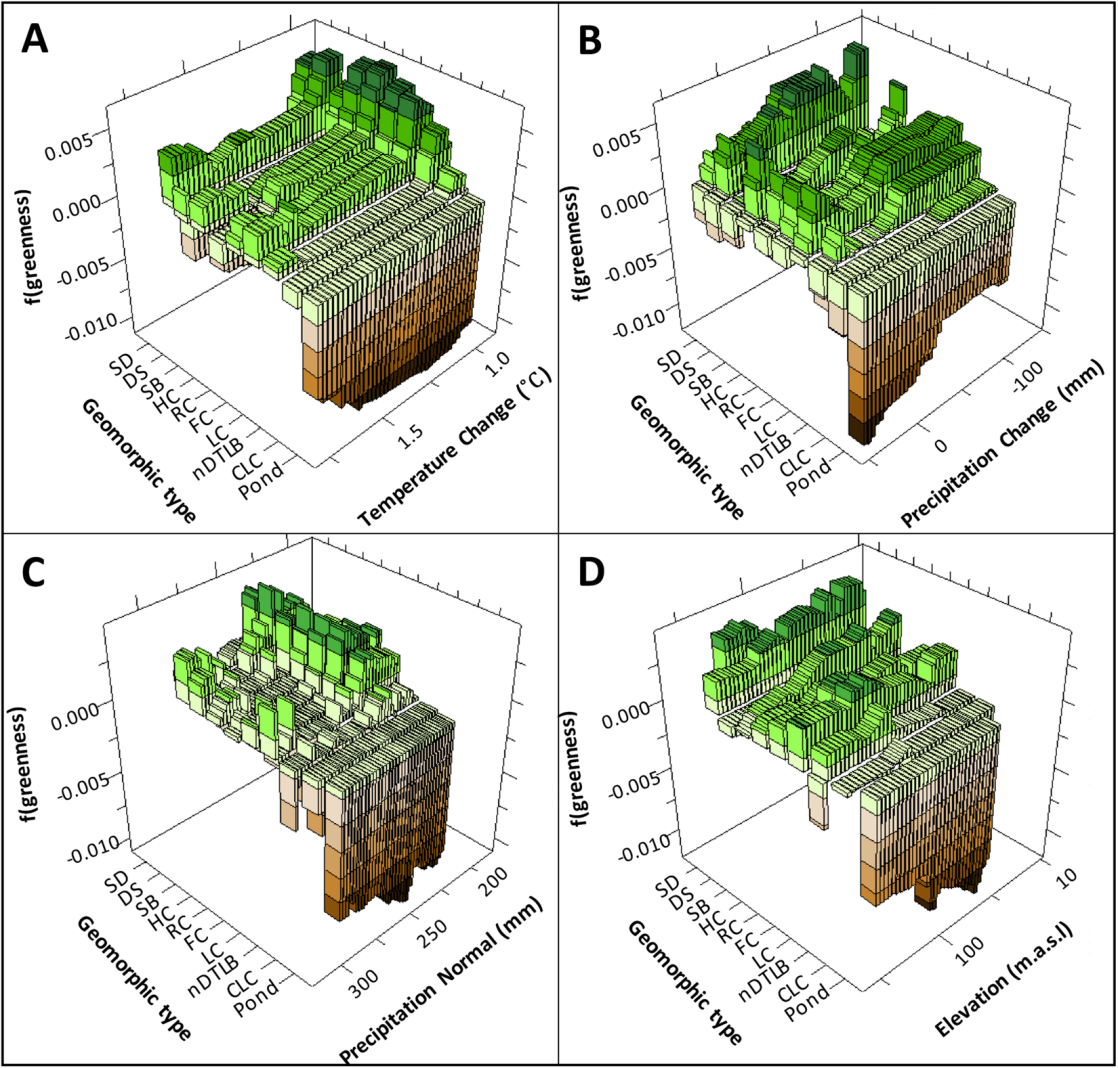
Three dimensional partial dependency plots from gradient boosting analysis, illustrating the strong interaction between geomorphic type and temperature change (**A**), precipitation change (**B**), precipitation normal (**C**), and elevation (**D**). Generally, green and brown colors indicate positive and negative NDVI trends, respectively. Geomorphic type acronyms correspond to sandy barren (SB), sand dune (SD), drainage slope (DS), high-center polygon (HC), flat-center polygon (FC), low-center polygon (LC), riparian corridors (RC), nonpatterned drained thaw lake basins (nDTLB), and coalescent low-center polygon (CLC). Figure created in Salford Systems TreeNet Salford Predictive Modeler v.8.0.

**Figure 4:**
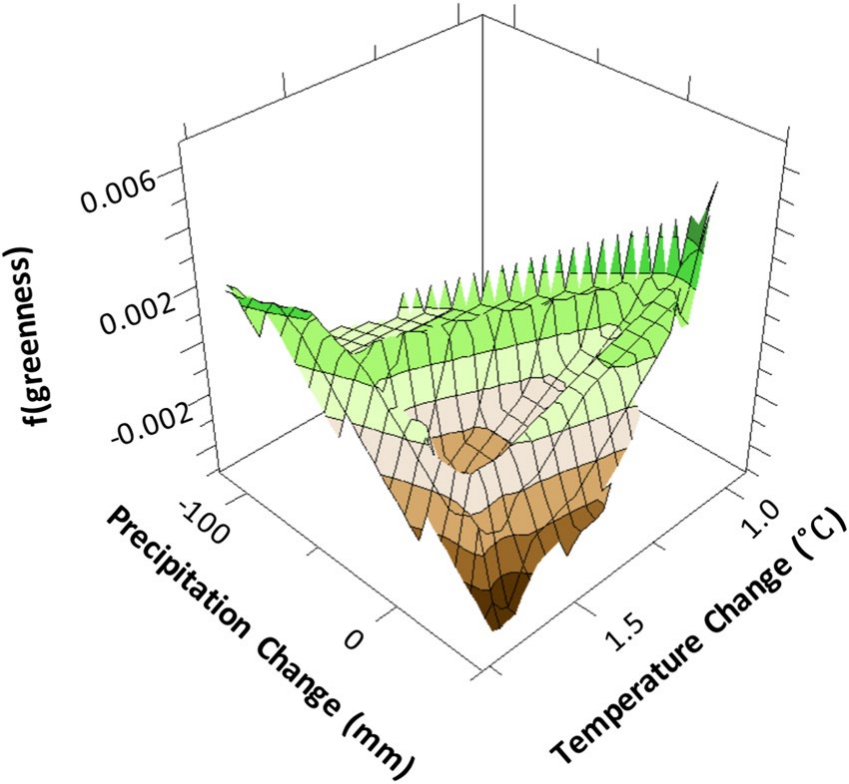
Three dimensional partial dependency plot from gradient boosting analysis, illustrating the impact both temperature change and precipitation change may have on NDVI trends. Figure created in Salford Systems TreeNet Salford Predictive Modeler v.8.0.

The gradient boosting analysis used 183 regression trees to construct a robust model that well represented the variability in historical greenness trends (learning/testing, R^2^ = 0.72, 0.62). After “shaving” or recursive predictor elimination procedures were complete, the final BRT analysis determined geomorphic type (100.0), temperature change (64.5), precipitation change (61.5), elevation (49.1), and precipitation normal (50.3) to be the most important factors (i.e. “variable importance”, expressed as a percentage, scaled to the most important factor) controlling greenness trends (Fig. 3, Supplemental Fig. S8). Partial dependency plots illustrate the strong control of geomorphic type on NDVI (Fig. 3, Supplemental Fig. S8), as generally, the higher the soil moisture the lower the rate of decadal greening. This is in line with partial dependency plots for precipitation and temperature change, which indicate that NDVI increased (i.e. greening) with reduced precipitation and cooler temperatures, whereas the NDVI decreased (i.e. browning) with increased precipitation and warmer temperatures (Fig. 4). Specifically, precipitation change greater than +31 mm was associated with substantial decreases in NDVI, while precipitation change associated with drier conditions, less than -10 mm increased NDVI. Interestingly, partial dependency plots for temperature change reveal that a potential NDVI threshold exists, inferring that if warming is limited to below +1.06 °C, the tundra continues to experience increased NDVI, but if warming exceeds that threshold NDVI is substantially reduced (Fig. 3, Supplemental Fig. S8). However, greening may slowly resume after +1.70 °C of warming. Although elevation and precipitation normal did not account for most of the BRT model variability, they nevertheless notably impacted tundra geomorphology greenness (Fig. 3). Because temperature and precipitation change predictors did not meet the assumption of normality, we used log transformed temperature and precipitation anomalies in the ensuing multivariate analysis.

Regionally, the cross-validated multivariate regression model identified climate anomalies of precipitation and temperature, wet%, and elevation to be the most important predictors of historical NDVI trends (R² = 0.49, p < 0.001; five-fold cross validation R² = 0.30), which suggests that increasing the decadal precipitation and temperature anomalies will decrease NDVI trends (i.e. browning), whereas an increase in wet% (i.e. nonpatterned drained thaw lake basins and low-center polygons) area will increase NDVI trends, both consistent with the BRT analysis. Furthermore, NDVI correlations with temperature and precipitation anomalies specific to geomorphic type, provide insight into regional scale controls on decadal NDVI change, as nearly all geomorphic types were sensitive to either temperature or precipitation anomalies (Table 2). Significant decreases in NDVI with warmer temperatures were found for high-center polygons, low-center polygons, sandy barrens, nonpatterned drained thaw lake basins, and riparian corridors, whereas pond NDVI increased with warming. Decreases in NDVI were also identified for sand dunes, drained slopes, and ponds associated with increased precipitation (Table 2). The geomorphic type most sensitive to temperature-mediated NDVI change, inferred from the highest decadal rate of change, were ponds (R² = 0.28, p = 0.002, n = 30) and riparian corridors (R² = 0.30, p = 0.001, n = 31), which increased and decreased NDVI with warming, respectively. In contrast, the geomorphic types most sensitive to precipitation-mediated reduction in the NDVI trend, were ponds (R² = 0.36, p < 0.001, n = 30), followed closely by drained slopes (R² = 0.22, p = 0.008, n = 31). We did not identify any significant relationships linking increased greening with increased precipitation among geomorphic types (Table 2), consistent with BRT analysis and regression model. We associated climate sensitivities to patterns of greenness in sandy barrens and sand dunes to spectral differences associated with saturated versus unsaturated soils in response to warmer/wetter conditions and not explicitly a vegetation response in these sparsely vegetated types (Table 2). Cumulatively, the relative importance of regional climate change for predicting the trajectory of greening versus browning was made strikingly apparent as ~61% or 35,800 km^2^ of the ACP were sensitive to change in temperature, whereas only ~10% or 5,900 km^2^ were sensitive to change in precipitation.

**Table 2:** Pearson correlation coefficients for geomorphic type and potential drivers of NDVI change. Positive and negative correlations indicate greening and browning, respectively, with increasing climate or elevation parameters. See Fig. 3 for geomorphic type definitions. Bolded = p ≤ 0.05; Italics = p ≤ 0.1.

Geomorphic Type	Temperature Anomaly	Precipitation Anomaly	Elevation
**SD**	0.13	**−0**.**43**	0.14
**DS**	−0.15	**−0**.**36**	−0.07
**SB**	−**0**.**58**	0.13	0.12
**HC**	−**0**.**42**	−0.07	−0.27
**RC**	−**0**.**48**	0.23	0.1
**FC**	−0.27	−0.13	−0.02
**LC**	−**0**.**49**	0.18	−0.18
**nDTLB**	−*0.31*	−0.19	0.03
**CLC**	−0.21	−0.21	0.17
**Pond**	**0**.**53**	−**0**.**60**	0.27

**Figure 5:**
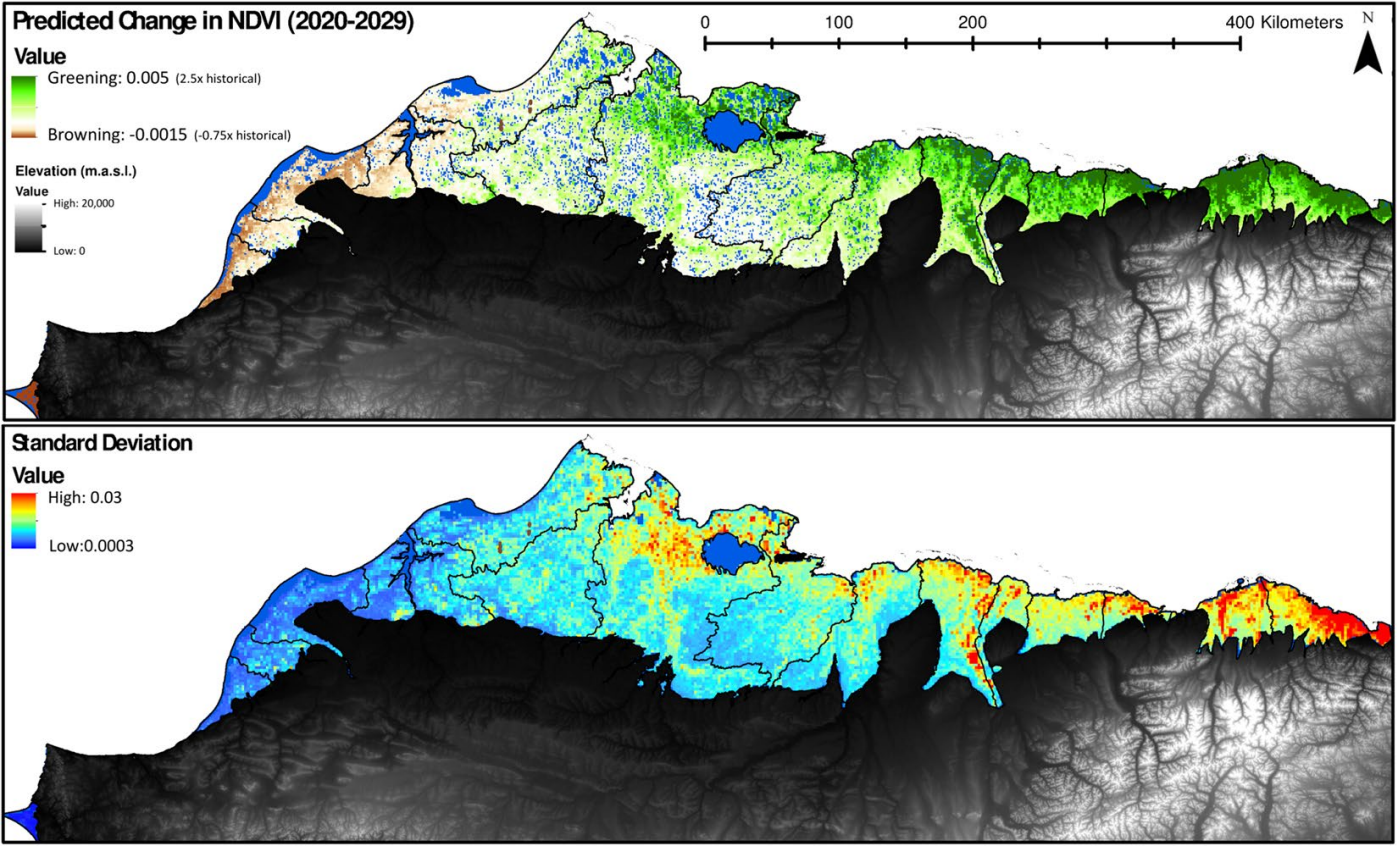
Forecasted change in greenness (2020–2029) relative to the long-term (1984–2014: +0.002) trend, using the five model mean and AR5 8.5 emission scenario (top panel). As a metric of future greenness uncertainty, the standard deviation is computed for all model outputs and emission scenarios (bottom panel). Maps created in Esri ArcMap 10.4.

**Figure 6:**
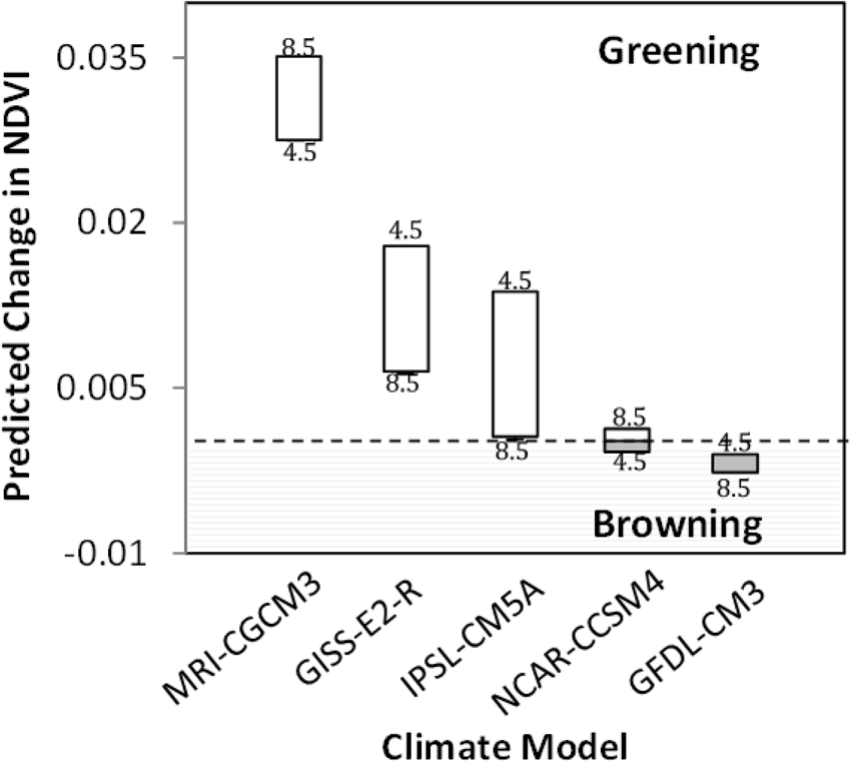
Projected variability in greenness (2020–2029) using five climate models and two emission scenarios. Above and below dotted line represent greening and browning relative to long-term trends, respectively, while RCP emission scenario highs and lows are indicated by 8.5 and 4.5, respectively.

To evaluate how NDVI trends may change over the next decade (i.e. 2020–2029), derived multivariate models were applied across the ACP using five IPCC climate models and two emission scenarios (i.e. RCP 8.5 and 4.5). Generally, we find the projected change in NDVI will likely vary in magnitude and spatial distribution (Fig. 5) associated with future climate change (Table 1). Simulations suggest the greatest magnitude of change in greening and browning relative to long term trends (1984–2014) were for the MRI-CGCM3 RCP 8.5 and GFDL-CM3 RCP 8.5 climates, respectively (Fig. 6). However, although, predicted change in NDVI notably varied between climate models, emission scenarios add another degree of uncertainty, as highlighted by the trajectory shift from greening to browning found with NCAR-CCSM4 and IPSL-CM5A (Fig. 6). Moreover, predicted change in NDVI may be expected to vary spatially (Fig. 5), as all models indicate the western Chukchi coast will experience the greatest browning (Fig. 5) and the northeastern Beaufort coastal plain will experience the most greening. However, regions anticipated to have the highest greening trends by 2020–2029, also have the highest uncertainty or disagreement among model outputs (Figs 5 and 6).

## Discussion

As the climate continues to warm, we can expect an increased occurrence of browning across the Arctic Coastal Plain of northern Alaska (Figs 2 and 5), but the rate at which the landscape browns, depends on the magnitude of temperature and precipitation change (Fig. 4), with the exception of young geomorphic types from recently drained lakes (e.g. nonpatterned drained thaw lake basins and low-center polygons) that will likely continue to green. These patterns were highlighted in our analysis as historical NDVI trends varied by tundra geomorphic type (Fig. 3). Browning was particularly sensitive to elevated temperature and/or precipitation (Table 2), both of which are predicted to increase across arctic tundra regions^31^.

To evaluate how NDVI might change over the next decade (2020–2029) we developed a predictive multivariate model using a range of decadal scale climate and geospatial data inputs (Supplemental Table S7), selected as important predictors in the BRT analysis. However, because of the decadal scale input datasets^32^ we are unable to explicitly and directly evaluate year-specific observations of browning (i.e. 2011)^11^ within this modeling framework. We still were able to provide insights into potential controls on greenness. Generally, it has been postulated that recent observations of browning, may have arisen from an array of annual/seasonal regional/site level changes that are largely correlated with decadal climate and land cover datasets, such as the delayed onset of the growing season and longer snow cover^32^, summer cloudiness^32^, winter warming^33,34^, or thermokarst development^9^. Model NDVI forecasting (Figs 5 and 6) identified greening to likely continue on the ACP, but will be highly dependent on the amplitude of future climate change (Fig. 6 and Table 1). Predicted NDVI by 2020–2029 was found to be variable dependent on climate model and RCP (Fig. 6), but the magnitude in greening versus browning was generally explained by the inverse relationship between temperature and NDVI change, as the greater the temperature change the lower the NDVI. Generally, all simulations find regional specific browning to occur on the southwestern Chukchi coast and greening to occur on the northeastern Beaufort coastal plains but the magnitudes vary by climate model (Fig. 6).

Trends in MaxNDVI estimated from Global Inventory Modeling and Mapping Studies (GIMMS) datasets^11^, were similar to that evaluated using Landsat imagery (Fig. 2), though, higher resolution imagery and newly developed maps enabled the evaluation of spatiotemporal heterogeneity in greenness, highlighting the strong interaction between landforms and climate influencing greenness trends (Table 2 and Fig. 3). We found trends in greenness were specific to geomorphic type and sensitive to either temperature or precipitation change (Table 2), with the exception of tundra ponds, which was found to be sensitive to both climatic drivers. The climatic sensitivity of tundra ponds is in line with hypothesized drivers of vegetation change^22,35,36^, as warmer temperatures may thaw permafrost and increase available nutrients in the water column^37,38^, potentially enabling the expansion of aquatic macrophytes^35,36^. Increased precipitation is likely to increase the ratio of standing water to macrophyte distribution in ponds, manifesting as an increased browning signal^9^. Although, relationships between decreasing NDVI and increasing temperature were identified for high-center polygons, low-center polygons, and riparian corridors (Table 2), it is important to note that significant greening occurred across all geomorphic types until temperatures warmed beyond +1.06 °C, where NDVI decreases sharply (Fig. 3, Supplemental Fig. S8). However, the greening trend may begin to increase if warming is greater than ~+1.70 °C. Generally, at the landscape scale, we find greening to increase if precipitation decreases more than -10 mm relative to normal precipitation patterns, whereas browning increased with precipitation. This pattern was likely identified in response to either wetting/drying of water saturated tundra soils and/or increased cloudiness (associated with increased precipitation) that may decrease productivity in response to a reduction in photosynthetically active radiation^32^. However, although NDVI generally decreased with increased precipitation, this pattern was not able to explain the regionally specific variability in NDVI that occurred among landforms (Fig. 3).

Although this work increases our understanding of past and future greenness patterns in arctic coastal tundra ecosystems, it is unclear if differences in greening versus browning trends for each geomorphic type (Table 2) were associated with local disturbances. For example, the prevalence of thermokarst pits (upland tundra: drained slopes and high-center polygons) and associated increases in surface wetness^7,39^, seasonally dependent patterns of herbivore activity (low-center polygons, nonpatterned drained thaw lake basins, and drained slopes)^40^, and/or plant community change (ponds, nonpatterned drained thaw lake basins, low-center polygons)^36^. The interpretation of greening and browning signals spanning small to large spatiotemporal scales (i.e plot to pan-Arctic) is difficult, as multiple climatic and environmental factors influence NDVI trends, which are likely related but spatially dependent. For example, at the Pan-Arctic scale, greening has been linked to extra-Arctic processes such as CO_2_ fertilization^41,42^, atmospheric nitrogen deposition^41^, as well as marine-terrestrial interactions related to ocean circulation patterns and sea ice decline^1,11^. At the landscape scale, other local processes such as the frequency and magnitude of wildfires^2^, regional climate change^1,2,11,41,42^, infrastructure development^9^, and shrubification^43^ are important. We find the distribution of tundra geomorphology (Fig. 3 and Table 2, Supplemental Fig. S2) is another major factor modulating greening and browning signals in ways previously not recognized. At the fine scale, processes related to changing patterns of phenology^44^, plant community change^22^, herbivory^29,45^ and surface hydrology driven by thermokarst^7,46^ can also notably impact vegetation productivity and NDVI. A holistic ecosystem perspective is required to unravel the spatiotemporal complexity involved with changing tundra greenness, which we are beginning to understand, but are limited by few observational data and comprehensive analyses across scales and platforms of observation.

Our findings indicate tundra geomorphic heterogeneity and regional climate change are dominant factors controlling decadal scale trends in greenness. Thus, a detailed understanding of how climate interacts with landforms is necessary for evaluating the spatiotemporal ecosystem interactions that impact regional-global patterns of plant productivity. Although, correlations between NDVI and vegetation productivity are robust across latitudinal gradients^47^, our findings have several implications for local controls on vegetation productivity in the expansive (~1.9 million km^2^)^24^ arctic coastal tundra ecosystem. Ecosystem and earth system models generally predict plant productivity to increase associated with projected climate change across northern latitudes. However, assuming our observed greenness trends correspond with productivity trends, we predict a reduction in carbon uptake potential across much of the ACP of northern Alaska in response to projected warmer and/or wetter climatic conditions. In combination, with deeper active layer depths^48^ exposing increased soil carbon to decomposition^48^, this further increases the potential for a net loss of carbon to the atmosphere, at a greater degree than previously expected. It is important to better understand how regional-global trends in greening and browning correspond to both local and regional phenomena to enhance our predictive capacity and ability to detect change in plant productivity across the Pan-Arctic to constrain our predictive uncertainty related to the future state and fate of global climate change^49^.

## Electronic supplementary material

Supplemental Information

## References

[1] Bhatt, U. S. *et al*. Circumpolar Arctic Tundra Vegetation Change Is Linked to Sea Ice Decline. *Earth Interact* **14** (2010).

[2] Goetz SJ, Bunn AG, Fiske GJ, Houghton RA (2005). Satellite-observed photosynthetic trends across boreal North America associated with climate and fire disturbance. Proc. Natl. Acad. Sci. USA.

[3] Jia, G. S. J., Epstein, H. E. & Walker, D. A. Greening of arctic Alaska, 1981–2001. *Geophysical Research Letters* **30** (2003).

[4] Myers-Smith, I. H. *et al*. Shrub expansion in tundra ecosystems: dynamics, impacts and research priorities. *Environ Res Lett* **6** (2011).

[5] Elmendorf SC (2012). Plot-scale evidence of tundra vegetation change and links to recent summer warming. Nat Clim Change.

[6] Bokhorst S, Bjerke JW, Street LE, Callaghan TV, Phoenix GK (2011). Impacts of multiple extreme winter warming events on sub-Arctic heathland: phenology, reproduction, growth, and CO2 flux responses. Global Change Biology.

[7] Liljedahl, A. K. *et al*. Pan-Arctic ice-wedge degradation in warming permafrost and its influence on tundra hydrology. *Nat Geosci* **9**, 312 (2016).

[8] Bhatt, U. S. *et al*. Implications of Arctic Sea Ice Decline for the Earth System. *Annual Review of Environment and Resources*, **39**, 57 (2014).

[9] Raynolds, M. K. & Walker, D. A. Increased wetness confounds Landsat-derived NDVI trends in the central Alaska North Slope region, 1985-2011. *Environ Res Lett* **11** (2016).

[10] Jorgenson MT, Grosse G (2016). Remote Sensing of Landscape Change in Permafrost Regions. Permafrost Periglac.

[11] Bhatt US (2013). Recent Declines in Warming and Vegetation Greening Trends over Pan-Arctic Tundra. Remote Sens-Basel.

[12] Phoenix GK, Bjerke JW (2016). Arctic browning: extreme events and trends reversing arctic greening. Global Change Biology.

[13] Nitze I, Grosse G (2016). Detection of landscape dynamics in the Arctic Lena Delta with temporally dense Landsat time-series stacks. Remote Sens Environ.

[14] Pattison RR, Jorgenson JC, Raynolds MK, Welker JM (2015). Trends in NDVI and Tundra Community Composition in the Arctic of NE Alaska Between 1984 and 2009. Ecosystems.

[15] Frost, G. V., Epstein, H. E. & Walker, D. A. Regional and landscape-scale variability of Landsat-observed vegetation dynamics in northwest Siberian tundra. *Environ Res Lett* **9** (2014).

[16] Emmerton CA (2016). Net ecosystem exchange of CO2 with rapidly changing high Arctic landscapes. Global Change Biology.

[17] McManus KM (2012). Satellite-based evidence for shrub and graminoid tundra expansion in northern Quebec from 1986 to 2010. Global Change Biology.

[18] Bartsch, A., Hofler, A., Kroisleitner, C. & Trofaier, A. M. Land Cover Mapping in Northern High Latitude Permafrost Regions with SatelliteData: Achievements and Remaining Challenges. *Remote Sens-Basel* 8 (2016).

[19] Lara MJ (2015). Polygonal tundra geomorphological change in response to warming alters future CO2 and CH4 flux on the Barrow Peninsula. Global Change Biology.

[20] Jorgenson, T. M. & Grunblatt, J. Landscape-Level Ecological Mapping of Northern Alaska and Field Site Photography (2013).

[21] Jorgenson, M. T. & Heiner, M. Ecosystems of northern Alaska. 1:2.5 million-scale map produced by ABR, Inc., Fairbanks, AK and the Nature Conservancy, Anchorage, AK. (2003).

[22] Lara, M. J. *et al*. Estimated change in tundra ecosystem function near Barrow, Alaska between 1972 and 2010. *Environ Res Lett* **7** (2012).

[23] USGS. A Watershed and Stream Hydrography Enhanced Dataset Project, Alaska Watersheds -5th Level, compiled by the Conservation Biology Institute. (2006).

[24] Walker DA (2005). The Circumpolar Arctic vegetation map. J Veg Sci.

[25] Kanevskiy M (2013). Ground ice in the upper permafrost of the Beaufort Sea coast of Alaska. Cold Reg Sci Technol.

[26] Lara, M. J., Nitze, I., Grosse, G. & McGuire, A. D. Tundra landform and vegetation productivity trend maps for the Arctic Coastal Plain of northern Alaska. *Scientific Data*, (in press). 10.1038/sdata.2018.58 PMC5892374 29633984

[27] Elith J, Leathwick JR, Hastie T (2008). A working guide to boosted regression trees. J Anim Ecol.

[28] SNAP. *Scenarios Network for Alaska and Arctic Planning*, *University of Alaska*. (2017).

[29] Lara MJ, Johnson DR, Andresen C, Hollister RD, Tweedie CE (2017). Peak season carbon exchange shifts from a sink to a source following 50+ years of herbivore exclusion in an Arctic tundra ecosystem. J Ecol.

[30] Walsh JE, Chapman WL, Romanovsky V, Christensen JH, Stendel M (2008). Global Climate Model Performance over Alaska and Greenland. J Climate.

[31] ACIA. Arctic climate impact assessment scientific report. *Cambridge University Press*, *Cambridge*, *UK*. (2005).

[32] Bieniek, P. A. *et al*. Climate Drivers Linked to Changing Seasonality of Alaska Coastal Tundra Vegetation Productivity. *Earth Interact* **19** (2015).

[33] Bokhorst SF, Bjerke JW, Tommervik H, Callaghan TV, Phoenix GK (2009). Winter warming events damage sub-Arctic vegetation: consistent evidence from an experimental manipulation and a natural event. J Ecol.

[34] Bokhorst S, Tommervik H, Callaghan TV, Phoenix GK, Bjerke JW (2012). Vegetation recovery following extreme winter warming events in the sub-Arctic estimated using NDVI from remote sensing and handheld passive proximal sensors. Environ Exp Bot.

[35] Andresen CG, Lougheed VL (2015). Disappearing Arctic tundra ponds: Fine-scale analysis of surface hydrology in drained thaw lake basins over a 65year period (1948–2013). Journal of Geophysical Research-Biogeosciences.

[36] Villarreal, S. *et al*. Tundra vegetation change near Barrow, Alaska (1972–2010). *Environ Res Lett* **7** (2012).

[37] Lougheed VL, Butler MG, McEwen DC, Hobbie JE (2011). Changes in Tundra Pond Limnology: Re-sampling Alaskan Ponds After 40 Years. Ambio.

[38] Reyes FR, Lougheed VL (2015). Rapid nutrient release from permafrost thaw in arctic aquatic ecosystems. Arct Antarct Alp Res.

[39] Jorgenson, M. T., Shur, Y. L. & Pullman, E. R. Abrupt increase in permafrost degradation in Arctic Alaska. *Geophysical Research Letters* **33** (2006).

[40] Batzli GO, Pitelka FA, Cameron GN (1983). Habitat Use by Lemmings near Barrow, Alaska. Holarctic Ecol.

[41] Zhu, Z. C. *et al*. Greening of the Earth and its drivers. *Nat Clim Change* **6**, 791 (2016).

[42] Los SO (2013). Analysis of trends in fused AVHRR and MODIS NDVI data for 1982–2006: Indication for a CO2 fertilization effect in global vegetation. Glob. Biogeochem. Cycle.

[43] Forbes BC, Fauria MM, Zetterberg P (2010). Russian Arctic warming and ‘greening’ are closely tracked by tundra shrub willows. Global Change Biology.

[44] de Jong R, de Bruin S, de Wit A, Schaepman ME, Dent DL (2011). Analysis of monotonic greening and browning trends from global NDVI time-series. Remote Sens Environ.

[45] Olofsson J, Tommervik H, Callaghan TV (2012). Vole and lemming activity observed from space. Nat Clim Change.

[46] Raynolds MK (2014). Cumulative geoecological effects of 62 years of infrastructure and climate change in ice-rich permafrost landscapes, Prudhoe Bay Oilfield, Alaska. Global Change Biology.

[47] Epstein, H. E. *et al*. Dynamics of aboveground phytomass of the circumpolar Arctic tundra during the past three decades. *Environ Res Lett* **7** (2012).

[48] Koven, C. D. *et al*. A simplified, data-constrained approach to estimate the permafrost carbon-climate feedback. *Philosophical Transactions of the Royal Society a-Mathematical Physical and Engineering Sciences* **373** (2015). 10.1098/rsta.2014.0423 PMC4608038 26438276

[49] Abbott, B. W. *et al*. Biomass offsets little or none of permafrost carbon release from soils, streams, and wildfire: an expert assessment. *Environ Res Lett* **11** (2016).

