# Supplementary material for: Reduced arctic tundra productivity linked with landform and climate change interactions
Source: Sci Rep. 2018 Feb 5;8:2345. doi: 10.1038/s41598-018-20692-8 (PMC5799341; doi:10.1038/s41598-018-20692-8)
Supplement: Supplementary file 1 — Supplemental Information [file 41598_2018_20692_MOESM1_ESM.doc]

# Reduced arctic tundra productivity linked with landform and climate change interactions

Mark J. Lara*, Ingmar Nitze, Guido Grosse, Philip Martin, and A. David McGuire

# Supplemental Information

Supplementary Table S1: Mosaicked Landsat scenes used to create the tundra geomorphology map

| Product ID | Sensor | Satellite | Year* | Month* | Day* |
| --- | --- | --- | --- | --- | --- |
| LC80690112013249LGN00 | OLI/TIRS | Landsat 8 | 2013 | Sept. | 5 |
| LC80720112013254LGN00 | OLI/TIRS | Landsat 8 | 2013 | Sept. | 10 |
| LC80740112014191LGN00 | OLI/TIRS | Landsat 8 | 2014 | July | 9 |
| LC80770102013193LGN00 | OLI/TIRS | Landsat 8 | 2013 | July | 11 |
| LC80770112013193LGN00 | OLI/TIRS | Landsat 8 | 2013 | July | 11 |
| LC80790102013191LGN00 | OLI/TIRS | Landsat 8 | 2013 | July | 9 |
| LC80800102014217LGN00 | OLI/TIRS | Landsat 8 | 2014 | Aug. | 4 |
| LC80800112014249LGN00 | OLI/TIRS | Landsat 8 | 2014 | Sept. | 5 |
| LC80820122013244LGN00 | OLI/TIRS | Landsat 8 | 2013 | Aug. | 31 |
| LC80830102014222LGN00 | OLI/TIRS | Landsat 8 | 2014 | Aug. | 9 |
| LC80830112014190LGN00 | OLI/TIRS | Landsat 8 | 2014 | July | 8 |
| LC80840122013194LGN00 | OLI/TIRS | Landsat 8 | 2013 | July | 12 |
| *Acquisition date |  | | | | |

Supplementary Table S2: Characteristics of dominant Alaskan Arctic Coastal Plain tundra geomorphic types

| **Geomorphic Type** | **Moisture Regime** | **Relief** | **Vegetation Community** |
| --- | --- | --- | --- |
| Coastal saline water | Open water | Low | vegetation absent |
| Lakes & River | Open water | Low | vegetation absent |
| Pond | Aquatic | Low | (i) Aquatic Arctophila, Carex, (ii) Dupontia graminoid , (iii) Seasonal short grass |
| Coalescent low-center | Aquatic | Intermediate | (i) Seasonally flooded Carex, Dupontia, Eriophorum graminoid tundra, (ii) Aquatic Arctophila, Carex, Dupontia graminoid tundra |
| Nonpatterned DTLB | Moist-Wet | Low | (i) Moist Carex, Poa, Luzula graminoid tundra, (ii) Wet Carex, Sphagnum graminoid tundra, (iii) Seasonally flooded Carex, Dupontia, Eriophorum graminoid |
| Low-center | Moist-Wet | Intermediate | (i) Moist Carex, Poa, Luzula graminoid tundra, (ii) Wet Carex, Sphagnum graminoid tundra, (iii) Seasonally flooded Carex, Dupontia, Eriophorum graminoid tundra |
| Sandy barren | Moist-Wet | Low | (i) Deciduous scrub |
| Flat-center | Dry-Moist | Intermediate | (i) Dry Arctagrostis, Luzula, Poa, Carex graminoid tundra, (ii) Moist Carex, Poa, Luzula graminoid tundra |
| Riparian corridors | Dry-Moist | Intermediate | (i) Deciduous dwarf scrub, (ii) Evergreen dwarf scru b |
| High-center | Dry | High | (i) Dry Arctagrostis, Luzula, Poa, Carex graminoid tundra |
| Drainage slope | Very Dry | Very high | (i) Dry Lichen Heath,(ii) Dry Arctagrostis, Luzula, Poa, Carex graminoid tundra |
| Sand dune | Very Dry | Very high | (i) Seasonal desert herb vegetation, (ii) Deciduous desert scrub |
|  | | | |
| DTLB = Drained Thaw Lake Basin | | |  |

Supplementary Table S3: Accuracy assessment represented as a confusion matrix. Bolded diagonal values within the matrix represent correctly identified pixels, where User and Producer accuracies are presented on the right vertical axis and bottom horizontal axis.

|  |  | **Reference Sites** | | | | | | | | | | | | |  |
| --- | --- | --- | --- | --- | --- | --- | --- | --- | --- | --- | --- | --- | --- | --- | --- |
|  | **Geomorphic type** | SB | SD | RC | DS | HC | FC | LC | nDTLB | CLC | Pond | River | Lake | CS | **User accuracy** |
| **Classification** | SB | **12** |  |  |  | 2 | 2 |  |  |  |  | 1 |  |  | 63% |
|  | SD | 3 | **12** |  |  | 2 |  |  |  |  |  |  |  |  | 71% |
|  | RC |  |  | **4** |  |  |  | 1 |  |  |  |  |  |  | 80% |
|  | DS |  |  |  | **50** | 19 | 4 |  |  |  |  |  |  |  | 69% |
|  | HC |  |  |  | 35 | **215** | 30 | 22 | 2 |  |  |  |  |  | 70% |
|  | FC |  |  |  |  | 11 | **32** | 3 |  |  |  |  |  |  | 71% |
|  | LC |  |  | 1 | 6 | 34 | 11 | **152** | 5 | 7 |  |  | 2 |  | 70% |
|  | nDTLB |  |  |  |  | 3 |  | 16 | **53** | 1 |  | 1 | 2 |  | 70% |
|  | CLC |  |  |  |  |  |  | 2 | 2 | **15** |  |  | 2 |  | 71% |
|  | Pond |  |  |  |  |  |  |  |  |  | **18** |  |  |  | 100% |
|  | River | 2 |  |  |  |  |  |  | 2 | 2 |  | **10** |  |  | 63% |
|  | Lake |  |  |  |  |  |  | 1 | 1 |  |  |  | **156** |  | 99% |
|  | CS |  |  |  |  |  |  |  |  |  |  |  | 1 | **28** | 97% |
|  | **Producer accuracy** | 71% | 100% | 80% | 55% | 75% | 41% | 77% | 82% | 56% | 100% | 83% | 96% | 100% | 1000 |
|  | **Overall accuracy** | **76%** |  |  |  |  |  |  |  |  |  |  |  |  |  |
|  | **Cohens Kappa** | **0.73** |  |  |  |  |  |  |  |  |  |  |  |  |  |

Supplementary Figure S4: Watershed specific patterns in NDVI with standard deviation bars (top panel) and percent cover of tundra geomorphic type (bottom panel).

Supplemental Table S5: Absolute change (1999-2014) in NDVI by geomorphic type within each watershed on the Alaskan Arctic Coastal Plains. Blank cells indicate the geomorphic type was not present in the associated watershed.

| HUC_8 | Low/ Upland | CLC | DS | FC | HC | LC | nDTLB | POND | RC | SB | SD |
| --- | --- | --- | --- | --- | --- | --- | --- | --- | --- | --- | --- |
| 19050405a | Low | 0.015 | 0.010 | 0.022 | 0.020 | 0.031 | 0.005 | -0.027 | 0.036 | 0.021 | 0.047 |
| 19050405b | Up | 0.010 | -0.001 | 0.019 | 0.020 | 0.043 | 0.037 | 0.000 | 0.036 | 0.014 | 0.031 |
| 19060101a | Low | 0.021 | 0.044 | 0.042 | 0.050 | 0.056 | 0.041 | -0.019 | 0.037 | 0.032 | 0.011 |
| 19060101b | Up | 0.021 | 0.044 | 0.042 | 0.050 | 0.056 | 0.041 | -0.019 | 0.037 | 0.032 | 0.011 |
| 19060102a | Low | 0.034 | 0.046 | 0.050 | 0.056 | 0.057 | 0.060 |  | 0.060 | 0.041 | 0.036 |
| 19060102b | Up | 0.039 | 0.043 | 0.048 | 0.050 | 0.060 | 0.050 |  | 0.048 | 0.033 | 0.045 |
| 19060103a | Low | -0.007 | 0.010 | 0.005 | 0.016 | 0.014 | -0.005 | -0.050 | 0.035 | 0.013 | 0.015 |
| 19060103b | Up | 0.036 | 0.025 | 0.032 | 0.031 | 0.043 | 0.032 | -0.006 | 0.047 | 0.026 | 0.055 |
| 19060201a | Low | 0.010 | 0.017 | 0.017 | 0.017 | 0.019 | 0.011 | -0.003 | 0.013 | 0.009 | 0.003 |
| 19060201b | Up | 0.013 | 0.018 | 0.018 | 0.022 | 0.020 | 0.008 | -0.011 | 0.005 | 0.014 | -0.006 |
| 19060202a | Low | 0.005 | 0.008 | 0.010 | 0.010 | 0.012 | 0.008 | -0.013 | 0.000 | 0.007 | 0.018 |
| 19060202b | Up | 0.013 | 0.013 | 0.021 | 0.023 | 0.021 | 0.013 | 0.005 | 0.020 | 0.026 | 0.006 |
| 19060203a | Low | -0.002 | 0.004 | 0.007 | 0.011 | 0.005 | 0.000 | -0.015 | 0.001 | 0.012 | 0.009 |
| 19060203b | Up | 0.007 | 0.014 | 0.017 | 0.021 | 0.014 | 0.006 | -0.010 | 0.010 | 0.006 | 0.008 |
| 19060204a | Low | 0.024 | 0.030 | 0.028 | 0.029 | 0.027 | 0.022 | 0.011 | 0.026 | 0.011 | 0.026 |
| 19060204b | Up | 0.019 | 0.028 | 0.028 | 0.025 | 0.024 | 0.017 | 0.002 | 0.022 | 0.021 | 0.027 |
| 19060205a | Low | 0.023 | 0.036 | 0.035 | 0.034 | 0.032 | 0.025 | 0.014 | 0.004 | 0.009 | 0.021 |
| 19060205b | Up | 0.014 | 0.023 | 0.022 | 0.019 | 0.019 | 0.012 | 0.000 | 0.016 | 0.009 | 0.023 |
| 19060303b | Up |  | 0.109 | 0.056 | -0.001 | 0.000 | -0.009 | -0.026 | 0.076 | 0.028 | 0.022 |
| 19060304a | Low | 0.006 | 0.040 | 0.029 | 0.023 | 0.029 | 0.019 | -0.004 | 0.011 | 0.018 | 0.040 |
| 19060304b | Up | 0.007 | 0.019 | 0.021 | 0.011 | 0.016 | 0.009 | -0.002 | 0.014 | 0.026 | 0.035 |
| 19060401a | Low | 0.022 | 0.049 | 0.040 | 0.039 | 0.036 | 0.026 | -0.002 | 0.018 | 0.009 | 0.021 |
| 19060401b | Up | 0.014 | 0.024 | 0.021 | 0.014 | 0.019 | 0.012 | 0.001 | 0.018 | 0.005 | 0.024 |
| 19060402a | Low | 0.015 | 0.031 | 0.022 | 0.024 | 0.024 | 0.015 | -0.021 | 0.019 | 0.003 | 0.015 |
| 19060402b | Up | 0.021 | 0.019 | 0.017 | 0.016 | 0.015 | 0.018 | 0.002 | 0.006 | 0.019 | 0.030 |
| 19060403a | Low | 0.029 | 0.043 | 0.034 | 0.041 | 0.034 | 0.037 | 0.008 | 0.040 | 0.022 | 0.043 |
| 19060403b | Up | 0.033 | 0.034 | 0.034 | 0.031 | 0.036 | 0.037 | 0.012 | 0.041 | 0.024 | 0.047 |
| 19060501a | Low | 0.019 | 0.051 | 0.012 | 0.027 | 0.019 | 0.029 | 0.023 | 0.007 | 0.011 | 0.016 |
| 19060501b | Up | 0.011 | 0.026 | -0.002 | 0.011 | 0.002 | 0.026 | 0.015 | 0.003 | 0.009 | 0.025 |
| 19060502a | Low | 0.030 | 0.064 | 0.062 | 0.056 | 0.049 | 0.036 | 0.001 | 0.019 | 0.018 | 0.041 |
| 19060502b | Up | 0.029 | 0.035 | 0.043 | 0.037 | 0.034 | 0.034 | 0.028 | 0.020 | 0.018 | 0.042 |
| 19060503a | Low | 0.004 | 0.041 | 0.022 | 0.025 | 0.005 | 0.008 | 0.009 | -0.013 | 0.021 | 0.031 |
| 19060503b | Up | 0.020 | 0.015 | 0.011 | 0.012 | -0.001 | 0.028 | 0.029 | 0.011 | 0.015 | 0.034 |

Supplemental Table S6: Percent change in NDVI by geomorphic type within each watershed on the Alaskan Arctic Coastal Plains. Blank cells indicate the geomorphic type was not present in the associated watershed.

| HUC_8 | Low/ Upland | CLC | DS | FC | HC | LC | nDTLB | POND | RC | SB | SD |
| --- | --- | --- | --- | --- | --- | --- | --- | --- | --- | --- | --- |
| 19050405a | Low | 3.4% | 2.5% | 4.5% | 4.1% | 6.1% | 1.7% | -3.7% | 6.9% | 4.4% | 8.8% |
| 19050405b | Up | 2.6% | 0.7% | 4.0% | 4.3% | 8.1% | 7.1% | 0.8% | 7.0% | 3.3% | 6.1% |
| 19060101a | Low | 4.5% | 8.3% | 7.9% | 9.3% | 10.3% | 7.8% | -2.4% | 7.1% | 6.3% | 2.6% |
| 19060101b | Up | 4.5% | 8.3% | 7.9% | 9.3% | 10.3% | 7.8% | -2.4% | 7.1% | 6.3% | 2.6% |
| 19060102a | Low | 6.6% | 8.6% | 9.3% | 10.4% | 10.6% | 11.0% |  | 11.0% | 7.8% | 7.0% |
| 19060102b | Up | 7.5% | 8.2% | 9.0% | 9.3% | 11.0% | 9.3% |  | 8.9% | 6.5% | 8.4% |
| 19060103a | Low | -0.4% | 2.6% | 1.7% | 3.5% | 3.3% | 0.0% | -7.7% | 6.8% | 3.0% | 3.3% |
| 19060103b | Up | 6.9% | 5.1% | 6.2% | 6.2% | 8.2% | 6.3% | -0.2% | 8.8% | 5.2% | 10.2% |
| 19060201a | Low | 2.6% | 3.6% | 3.7% | 3.7% | 4.0% | 2.7% | 0.4% | 3.0% | 2.4% | 1.4% |
| 19060201b | Up | 3.0% | 3.8% | 3.9% | 4.6% | 4.2% | 2.3% | -1.0% | 1.6% | 3.3% | -0.1% |
| 19060202a | Low | 1.7% | 2.2% | 2.6% | 2.6% | 2.8% | 2.3% | -1.3% | 0.9% | 2.0% | 3.8% |
| 19060202b | Up | 3.1% | 3.1% | 4.4% | 4.8% | 4.4% | 3.0% | 1.7% | 4.2% | 5.3% | 1.9% |
| 19060203a | Low | 0.5% | 1.6% | 2.0% | 2.7% | 1.8% | 0.8% | -1.7% | 1.0% | 2.9% | 2.4% |
| 19060203b | Up | 2.0% | 3.3% | 3.6% | 4.3% | 3.2% | 1.9% | -0.8% | 2.6% | 1.9% | 2.3% |
| 19060204a | Low | 4.9% | 5.9% | 5.6% | 5.7% | 5.5% | 4.6% | 2.7% | 5.2% | 2.7% | 5.3% |
| 19060204b | Up | 4.0% | 5.6% | 5.6% | 5.1% | 5.0% | 3.7% | 1.2% | 4.6% | 4.4% | 5.4% |
| 19060205a | Low | 4.8% | 7.0% | 6.7% | 6.6% | 6.2% | 5.1% | 3.3% | 1.6% | 2.3% | 4.4% |
| 19060205b | Up | 3.2% | 4.8% | 4.6% | 4.1% | 4.0% | 2.9% | 0.9% | 3.5% | 2.4% | 4.7% |
| 19060303b | Up | 0.8% | 19.2% | 10.3% | 0.6% | 0.9% | -0.7% | -3.6% | 13.8% | 5.6% | 4.6% |
| 19060304a | Low | 1.8% | 7.7% | 5.8% | 4.8% | 5.8% | 4.0% | 0.2% | 2.8% | 3.9% | 7.6% |
| 19060304b | Up | 2.1% | 4.1% | 4.3% | 2.6% | 3.5% | 2.4% | 0.4% | 3.3% | 5.2% | 6.7% |
| 19060401a | Low | 4.5% | 9.2% | 7.6% | 7.5% | 7.0% | 5.3% | 0.4% | 3.8% | 2.3% | 4.4% |
| 19060401b | Up | 3.2% | 4.9% | 4.4% | 3.2% | 4.0% | 2.8% | 1.1% | 3.9% | 1.7% | 4.8% |
| 19060402a | Low | 3.5% | 6.1% | 4.5% | 5.0% | 4.9% | 3.5% | -2.7% | 4.0% | 1.4% | 3.4% |
| 19060402b | Up | 4.5% | 4.1% | 3.7% | 3.5% | 3.4% | 3.9% | 1.2% | 1.8% | 4.0% | 5.8% |
| 19060403a | Low | 5.8% | 8.1% | 6.7% | 7.8% | 6.6% | 7.1% | 2.2% | 7.6% | 4.5% | 8.2% |
| 19060403b | Up | 6.5% | 6.6% | 6.7% | 6.1% | 6.9% | 7.2% | 2.8% | 7.8% | 5.0% | 8.8% |
| 19060501a | Low | 4.0% | 9.4% | 2.8% | 5.3% | 4.0% | 5.7% | 4.7% | 2.1% | 2.7% | 3.5% |
| 19060501b | Up | 2.7% | 5.3% | 0.5% | 2.6% | 1.2% | 5.2% | 3.4% | 1.3% | 2.3% | 5.2% |
| 19060502a | Low | 6.0% | 11.7% | 11.3% | 10.3% | 9.1% | 7.0% | 0.9% | 4.1% | 3.8% | 7.7% |
| 19060502b | Up | 5.7% | 6.8% | 8.2% | 7.1% | 6.5% | 6.6% | 5.6% | 4.2% | 4.0% | 8.0% |
| 19060503a | Low | 1.5% | 7.8% | 4.6% | 5.2% | 1.6% | 2.2% | 2.4% | -1.4% | 4.3% | 6.1% |
| 19060503b | Up | 4.3% | 3.3% | 2.8% | 2.8% | 0.7% | 5.5% | 5.8% | 2.7% | 3.3% | 6.5% |

Supplemental Table S7: Summary of parameters used in multiple regression analysis. Variables Tnorm, Tdiff, Pnorm, Pdiff, Elev, represent the long term temperature normal (ᵒC), temperature change (ᵒC), precipitation normal (mm), precipitation change (mm), and elevation (m). See methods for soil moisture details.

| HUC_8 | Low/ Upland | NDVI trend | NDVI% change | Tnorm | Tdiff | Pnorm | Pdiff | Elev | Dry% | Moist% | Wet% | Aqua% | Water% | Other% |
| --- | --- | --- | --- | --- | --- | --- | --- | --- | --- | --- | --- | --- | --- | --- |
| 19050405a | Low | 0.0147 | 2.9% | -6.669 | 1.032 | 286.99 | 17.58 | 20.78 | 65.1% | 2.8% | 20.0% | 0.7% | 9.4% | 2.0% |
| 19060101a | Low | 0.0459 | 7.8% | -9.973 | 0.964 | 265.51 | 27.44 | 40.16 | 70.4% | 2.6% | 21.9% | 1.6% | 1.9% | 1.7% |
| 19060102a | Low | 0.0535 | 9.8% | -10.04 | 0.954 | 238.65 | 26.23 | 43.66 | 41.6% | 7.2% | 44.6% | 1.6% | 2.8% | 2.2% |
| 19060103a | Low | 0.0105 | 2.0% | -10.48 | 1.058 | 243.74 | 28.48 | 34.9 | 38.3% | 9.9% | 41.3% | 2.8% | 5.7% | 1.9% |
| 19060201a | Low | 0.0168 | 3.1% | -11.1 | 1.355 | 240.6 | 39.27 | 48.9 | 53.6% | 9.6% | 32.3% | 2.2% | 2.0% | 0.3% |
| 19060202a | Low | 0.0098 | 1.9% | -11.7 | 1.634 | 214.57 | 36.58 | 34.99 | 30.7% | 6.0% | 52.3% | 4.9% | 5.8% | 0.3% |
| 19060203a | Low | 0.0062 | 1.2% | -11.89 | 1.685 | 224.45 | 38.97 | 44.4 | 29.0% | 5.4% | 51.5% | 4.8% | 7.5% | 1.9% |
| 19060204a | Low | 0.0261 | 5.6% | -12.05 | 1.657 | 225.27 | 20.04 | 21.31 | 22.1% | 5.1% | 57.0% | 6.5% | 7.3% | 1.9% |
| 19060205a | Low | 0.0317 | 6.5% | -11.92 | 1.658 | 224.08 | -3.44 | 29.18 | 38.9% | 6.0% | 45.0% | 2.8% | 7.0% | 0.4% |
| 19060304a | Low | 0.026 | 5.9% | -11.73 | 1.618 | 214.84 | -13.3 | 29.43 | 41.6% | 7.9% | 37.2% | 2.4% | 5.7% | 5.1% |
| 19060401a | Low | 0.0368 | 8.6% | -11.8 | 1.627 | 214.93 | -19.5 | 37.13 | 29.8% | 9.9% | 47.7% | 4.9% | 5.2% | 2.6% |
| 19060402a | Low | 0.0181 | 6.2% | -11.66 | 1.629 | 221.92 | -26.4 | 35.31 | 28.1% | 16.6% | 31.4% | 7.3% | 5.5% | 11.1% |
| 19060403a | Low | 0.0372 | 8.0% | -11.66 | 1.645 | 234.16 | -41.3 | 29.24 | 48.0% | 16.4% | 26.2% | 5.8% | 2.0% | 1.5% |
| 19060501a | Low | 0.0232 | 5.2% | -11.66 | 1.679 | 254.92 | -65.3 | 22.97 | 51.3% | 14.0% | 23.7% | 4.4% | 2.9% | 3.7% |
| 19060502a | Low | 0.0531 | 10.1% | -11.67 | 1.748 | 284.63 | -108 | 34.68 | 46.9% | 17.2% | 30.5% | 1.4% | 1.0% | 2.9% |
| 19060503a | Low | 0.0225 | 4.9% | -11.56 | 1.598 | 267.57 | -95.3 | 32.86 | 49.1% | 17.6% | 28.0% | 1.3% | 0.9% | 3.1% |
| 19060303b | Up | 0.0081 | 3.2% | -11.45 | 1.5 | 240.19 | -9.94 | 158.9 | 65.9% | 3.0% | 10.3% | 0.3% | 8.7% | 11.7% |
| 19060304b | Up | 0.0132 | 2.6% | -11.69 | 1.556 | 226.36 | -11.7 | 159.7 | 65.1% | 3.7% | 26.3% | 1.3% | 2.9% | 0.7% |
| 19060401b | Up | 0.0166 | 3.7% | -11.32 | 1.561 | 218.74 | -17.4 | 208.9 | 43.1% | 7.2% | 44.8% | 2.0% | 2.3% | 0.7% |
| 19060402b | Up | 0.0159 | 4.8% | -11.3 | 1.553 | 223.7 | -21 | 216.5 | 44.9% | 15.7% | 27.6% | 3.2% | 3.3% | 5.3% |
| 19060403b | Up | 0.0324 | 6.4% | -11.56 | 1.602 | 240.41 | -38.9 | 188.8 | 60.7% | 11.1% | 23.2% | 2.8% | 1.2% | 0.9% |
| 19060501b | Up | 0.01 | 3.4% | -11.57 | 1.64 | 255.37 | -60.2 | 163.6 | 64.5% | 11.7% | 11.5% | 0.7% | 1.0% | 10.7% |
| 19060502b | Up | 0.0367 | 7.0% | -11.15 | 1.732 | 297.35 | -114 | 345.4 | 55.8% | 15.7% | 25.3% | 0.8% | 0.3% | 2.1% |
| 19060503b | Up | 0.0114 | 2.5% | -11.2 | 1.647 | 280.61 | -104 | 262.3 | 63.0% | 16.4% | 17.7% | 0.7% | 0.2% | 2.1% |
| 19050405b | Up | 0.0053 | 0.9% | -6.81 | 1.022 | 288.75 | 18.01 | 99.39 | 89.3% | 0.7% | 6.8% | 0.7% | 1.8% | 0.7% |
| 19060101b | Up | 0.0405 | 6.5% | -10.65 | 0.979 | 270.54 | 28.97 | 98.68 | 71.0% | 1.6% | 25.6% | 0.3% | 1.3% | 0.1% |
| 19060102b | Up | 0.0509 | 8.7% | -11.14 | 1.035 | 253.25 | 29.55 | 104.4 | 51.5% | 2.3% | 42.0% | 1.0% | 2.7% | 0.5% |
| 19060103b | Up | 0.0325 | 5.8% | -10.95 | 1.115 | 246.75 | 31.17 | 114 | 59.7% | 5.0% | 32.1% | 1.2% | 1.0% | 0.9% |
| 19060201b | Up | 0.019 | 3.4% | -11.36 | 1.396 | 242.85 | 41.79 | 98.99 | 39.8% | 7.5% | 47.1% | 3.6% | 2.0% | 0.0% |
| 19060202b | Up | 0.0199 | 4.0% | -11.6 | 1.568 | 223.78 | 41.05 | 75.89 | 29.5% | 6.5% | 56.8% | 4.5% | 2.7% | 0.0% |
| 19060203b | Up | 0.0162 | 2.9% | -11.87 | 1.629 | 234.99 | 42.76 | 98.78 | 44.8% | 3.7% | 45.5% | 2.6% | 3.3% | 0.1% |
| 19060204b | Up | 0.0242 | 4.7% | -11.95 | 1.564 | 241.61 | 20.29 | 156.7 | 42.4% | 6.1% | 41.9% | 3.7% | 4.7% | 1.1% |
| 19060205b | Up | 0.0187 | 3.5% | -11.86 | 1.582 | 232.67 | -0.72 | 162.2 | 52.8% | 5.0% | 34.1% | 2.6% | 5.0% | 0.5% |

Supplemental Figure S8: Two dimensional partial dependency plots, illustrating the effect of Land cover, Temperature change, Precipitation change, Precipitation normal, and elevation on surface greenness.
